# Normalized Protein Catabolic Rate Is a Superior Nutritional Marker Associated With Dialysis Adequacy in Continuous Ambulatory Peritoneal Dialysis Patients

**DOI:** 10.3389/fmed.2020.603725

**Published:** 2021-01-12

**Authors:** Aiya Qin, Xiang Liu, Xiaomeng Yin, Huan Zhou, Yi Tang, Wei Qin

**Affiliations:** ^1^Division of Nephrology, Department of Medicine, West China Hospital, Sichuan University, Chengdu, China; ^2^West China School of Medicine, Sichuan University, Chengdu, China

**Keywords:** continuous ambulatory peritoneal dialysis (CAPD), normalized protein catabolic rate (nPCR), nutritional status, dialysis adequacy, inadequate dialysis

## Abstract

**Introduction:** Current knowledge of the relationship between normalized protein catabolic rate (nPCR) and dialysis adequacy is limited. Our study aimed to explore the potential relationship between nPCR and dialysis adequacy.

**Methods:** In this cross-sectional study, we analyzed the association of nPCR with peritoneal dialysis adequacy in 266 continuous ambulatory peritoneal dialysis (CAPD) patients (mean age 48.6 ± 13.1 years; 50.8% male). The patients were divided into two groups: a dialysis inadequacy group (total weekly Kt/V urea < 1.70) and a dialysis adequacy group (total weekly Kt/V urea≥1.70). We then analyzed the correlation between dialysis adequacy and the patients' primary cause of end-stage renal disease, nutritional and inflammatory markers, and biochemical parameters. Multivariable logistic regression analysis was also used to identify risk factors for inadequate dialysis.

**Results:** We observed a significantly higher level of nPCR (0.98 ± 0.22 vs. 0.79 ± 0.18 g/kg/day, *p* < 0.001) in the dialysis adequacy group, whereas we observed no significant differences among other nutritional markers such as albumin, prealbumin, and transferrin. Correlation analyses revealed that dialysis adequacy was positively associated with residual glomerular filtration rate (rGFR), hemoglobin, serum calcium, and body mass index (BMI), while dialysis adequacy was negatively associated with leak-protein, uric acid, high-sensitivity C-reactive protein, interleukin-6, and serum phosphorus. Furthermore, a logistic regression analysis revealed that gender (male), nPCR <0.815 g/kg/day, higher weight, and rGFR <2.43 mL/min/1.73 m^2^ were independent risk factors for inadequate dialysis.

**Conclusion:** Nutritional status is closely associated with dialysis adequacy. Among common nutritional markers, nPCR may be superior for predicting CAPD dialysis adequacy. Gender (male), nPCR <0.815 g/kg/day, higher weight, and rGFR <2.43 mL/min/1.73 m^2^ are independent risk factors for dialysis inadequacy in CAPD patients.

## Introduction

Presently there are more than 40,000 peritoneal dialysis (PD) patients in China, representing ~20% of the total dialysis population. Dialysis adequacy, usually assessed by total urea clearance (Kt/V), is recognized as a crucial factor affecting prognosis in PD patients. Inadequate dialysis leads to higher morbidity, technical failure, and mortality (1, 2). It has been reported that nutritional status may also be related to the survival rate of PD patients. However, studies examining the relationship between nutritional markers and dialysis adequacy have reported inconsistent results. For example, it was previously reported that dialysis adequacy is influenced by blood pressure, anemia, nutrition status, inflammatory status, and electrolyte balance (3), while another study failed to demonstrate any association between Kt/V urea and nutrition status (4). Further study is therefore needed to determine whether one or more nutritional markers can predict PD adequacy.

Indeed, malnutrition is highly prevalent in dialysis patients, and a major contributor to morbidity and mortality, whereas the assessment of nutritional status itself is fraught with difficulties and remains controversial (5). Traditional markers of malnutrition such as serum albumin (Alb), prealbumin (PA), hemoglobin (Hb), transferrin (TRF), and body mass index (BMI) are influenced not only by nutritional status but also by age, gender, inflammation status, and liver function (6). These markers may therefore not be sensitive enough to predict dialysis adequacy. Normalized protein catabolic rate (nPCR), on the other hand, more accurately monitors protein intake, and has being increasingly advocated as an advantageous measure for nutritional status with possible prognostic importance in dialysis patients (7). Poor nutritional status is generally defined as nPCR <0.8 g/kg/day by the KDOQI guidelines and other authorities (5, 8). The goal of this study was to clarify the potential predictive factors (especially nPCR) associated with dialysis adequacy in Chinese PD patients.

## Materials and Methods

### Study Design and Participants

This cross-sectional study was carried out in the peritoneal dialysis units within the West China Hospital of Sichuan University. The study protocol was approved by the Medical Ethical Committee of West China Medical School, Sichuan University (FF-33-2019). A total of 316 patients were screened for inclusion in the study. Patients with CAPD duration of <3 months often suffer from more severe uremic toxins which might influence dialysis adequacy as well as the veracity of nPCR. So, in medical research, nPCR is often evaluated 3 months after CAPD. Fifty patients were excluded for the following reasons: age under 18 years, CAPD duration <3 months, experienced a peritonitis episode within the previous 30 days, or their medical record lacked important study data. All patients included in this study received glucose-based, lactate-buffered dialysis solutions (Dianeal, Baxter, Guangzhou, China) and were prescribed 2-L bags of dialysate containing 1.5 or 2.5% dextrose, with 3–5 exchanges per day. Written informed consent was obtained from every participant.

### Clinical Data Collection

Patient demographic features such as age, gender, body weight, height, primary renal disease, and duration of CAPD were collected. Serum albumin (Alb), prealbumin (PA), serum creatinine (Scr), triglycerides (TG), cholesterol (TC), uric acid (UA), transferrin (TRF), high-sensitivity C-reactive protein (hsCRP), interleukin-6 (IL-6), intact parathyroid hormone (iPTH), hemoglobin (Hb), serum calcium (Ca), and serum phosphorus (P) were measured in all patients.

### Peritoneal Equilibrium Test (PET)

In accordance with the ISPD guidelines (1), a standard PET was performed: dialysate samples were collected at 0, 2, and 4 h, and a blood sample was collected at 120 min. All blood and dialysate samples were analyzed within 24 h of collection. According to the 4-h dialysate-to-plasma ratio for creatinine (D/Pcr), peritoneal solute transport was classified as: low (L) when D/Pcr < 0.50; low-average (LA) when D/Pcr was 0.50–0.65; high-average (HA) when D/Pcr was 0.65–0.82; and high (H) when D/Pcr >0.82. Residual glomerular filtration rate (rGFR) = (renal urea clearance +renal creatinine clearance)/2, where renal urea clearance (ml/min) = (urine urea concentration/serum urea concentration) × 24 h urine volume/1,440, and renal creatinine clearance (ml/min) = (urine creatinine concentration/serum creatinine concentration) × 24 h urine volume/1,440. The total protein leak per day (leak-protein) was calculated based on the determination of D24h-Protein and protein loss from urine in the last 24 h.

### Dialysis Adequacy

Based on the KDOQI and ISPD guidelines (1, 9), inadequate dialysis was defined as total weekly Kt/V urea <1.70. Total weekly Kt/V urea was calculated by the sum of peritoneal Kt/V urea (calculated from 24-h peritoneal dialysate) and renal Kt/V urea (calculated from 24-h urine) using the formula recommended in the KDOQI guidelines (9).

### Nutritional and Inflammatory Markers

The nutritional assessment markers used in our study included Alb, PA, TRF, BMI, nPCR, TG, and TC. BMI was calculated as weight/height^2^ (kg/m^2^). Overweight was defined as BMI ≥ 24.0 kg/m^2^. nPCR was calculated by the formula proposed by Tattersall (10), as follows: nPCR g/kg/day = 149.7 × G/V + 0.17, where G/V = [(U_v_ × U_c_) + (D_v_ × D_c_)]/1,440 V (U_v_ = volume of 24 h urine collection; U_c_ = urea concentration in 24 h urine collection; D_v_ = volume of 24 h collection of spent dialysate; D_c_ = urea concentration in 24 h collection of spent dialysate). hsCRP and IL-6 were included as markers of inflammation.

### Statistical Analysis

Patient characteristics were described by means, SD, medians, interquartile ranges, counts, and percentages as dictated by data type. Between-group comparisons were performed using Student's *t*-test, one-way analysis of variance, Kruskal-Wallis or chi-square tests as appropriate. Correlations were calculated using the Spearman correlation coefficient. Logistic regression was used to estimate the odds ratio of dialysis inadequacy associated with individual serum biochemical parameters and other dialysis parameters. Receiver-operating characteristic (ROC) analysis, with an estimate of the area under the ROC curve (AUC), was used to identify significant predictors of dialysis adequacy. All of these data were analyzed using the SPSS 23.0 software package (SPSS, Chicago, IL, USA) and *p*-values were calculated as two-sided. Statistical significance was set at *p* < 0.05.

## Results

### Demographic Characteristics

**Figure 1:**
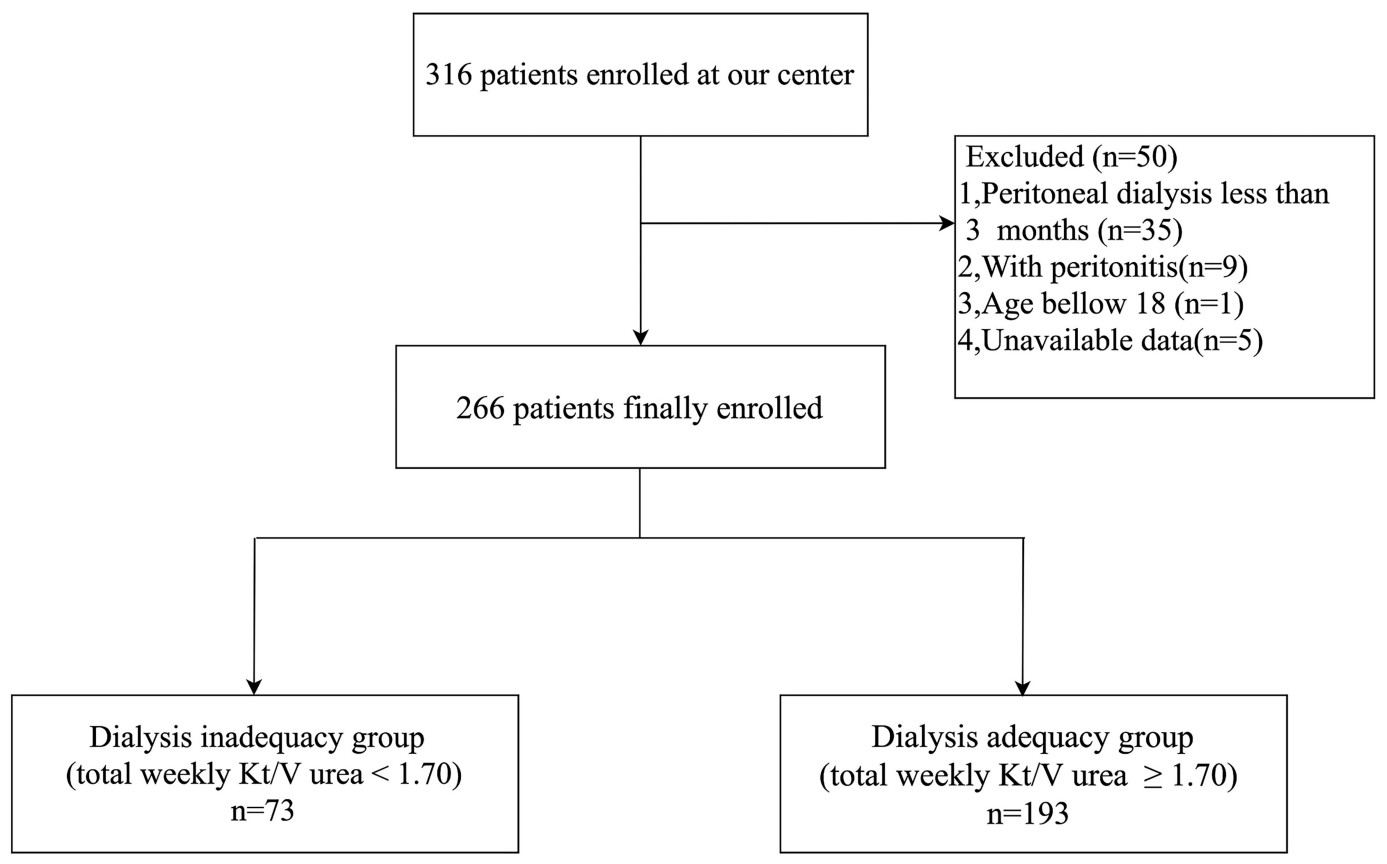
Study flow of participants.

Among the 316 CAPD patients reviewed for inclusion in this study, 50 patients were excluded and 266 patients were included in the final analysis (Figure 1). Glomerulonephritis (54.1%) was the most common primary cause of end-stage renal disease (ESRD), followed by hypertensive nephrosclerosis (20.0%), and diabetes mellitus (DM) (13.2%). According to their total weekly Kt/V urea, patients were divided into the dialysis inadequacy group (*n* = 73, 27.8%) or the dialysis adequacy group (*n* = 193, 72.2%). The characteristics of the study population, primary cause of ESRD, peritoneal transport type, and dialysis adequacy markers are summarized in Table 1. There were significant differences in gender, the primary cause of ESRD, and peritoneal transport type between the groups. Patients with high-average transport status had a marginally higher proportion of inadequate dialysis compared with patients with low-average status (44.6 vs. 25.4%, *p* = 0.057), while there were no statistically significant differences between the other subgroups (*p* > 0.1). The inadequate dialysis group included a significantly greater number of DM cases (24.7 vs. 8.8%, *p* = 0.002), while the dialysis adequacy group had a significantly higher proportion of glomerulonephritis patients (58.5 vs. 42.5%, *p* = 0.020). In addition, dialysis adequacy was positively associated with rGFR (*p* < 0.001) and negatively associated with both Scr (*p* < 0.001) and leak-protein levels (*p* < 0.001). As for markers of mineral and bone disorder, we observed significantly higher serum phosphorus (*p* < 0.001) and lower serum calcium (*p* < 0.001) in the dialysis inadequacy group. There were no significant differences between the two groups in terms of age, PD time, or iPTH level.

**Table 1:** Demographic characteristics of CAPD patients.

**Patient characteristics**	**Total**	**Dialysis inadequacy group**	**Dialysis adequacy group**	***p-*value**
No.	266	73 (27.8%)	193 (72.2%)	
Gender (M/FM)	135/131	60/13	75/118	<0.001*
Age (years)	48.6 ± 13.1	50.9 ± 14.7	47.8 ± 12.4	0.085
Time on PD (months)	24 (11–51)	24 (14–45)	23 (8–53)	0.782
**Primary cause of ESRD**				
DM [*n* (%)]	35 (13.2)	18 (24.7)	17 (8.8)	0.002*
HTN [*n* (%)]	53 (20.0)	16 (21.9)	37 (19.2)	0.610
GN [*n* (%)]	144 (54.1)	31 (42.5)	113 (58.5)	0.020*
Other [*n* (%)]	34 (12.7)	8 (10.9)	26 (13.5)	–
Peritoneal transport type				0.024*
High [*n* (%)]	71 (26.7)	20 (27.4)	51 (26.4)	0.659
HA [*n* (%)]	130 (48.9)	44 (60.3)	86 (44.6)	0.057^#^
LA [*n* (%)]	58 (24.4)	9 (12.3)	49 (25.4)	1.000
L [*n* (%)]	7 (2.6)	0 (0.0)	7 (3.6)	0.670
Scr (μmol/L)	912.0 (726.0–1,139.0)	1,150.0 (938.5–1,145.0)	841.5 (673.5–1,043.7)	<0.001*
Kt/V	2.02 ± 0.50	1.46 ± 0.17	2.23 ± 0.44	<0.001*
rGFR (mL/min/1.73 m^2)^	1.31 (0–3.69)	0.51 (0–1.57)	1.90 (0–4.42)	<0.001*
leak-Protein (g/day)	5.72 (4.33–7.38)	6.16 (4.70–8.47)	5.43 (4.02–7.17)	0.023*
Ca (μmol/L)	2.3 ± 0.2	2.1 ± 0.2	2.2 ± 0.2	0.007*
P (μmol/L)	1.6 ± 0.5	1.8 ± 0.5	1.5 ± 0.5	<0.001*
iPTH (pmol/L)	26.0 (13.6–42.9)	31.6 (18.7–43.1)	24.5 (12.3–42.8)	0.057

Data presented as median (first-third interquartile range) or mean ±SD or number (percentage). *Stands for p < 0.05 M = male; ^#^stands for p < 0.1 compared to low-average group.

*DM, diabetes mellitus; HTN, hypertensive nephrosclerosis; GN, glomerulonephritis; L, low; LA, low average; HA, high average; H, high; FM, female; D/P Cr, dialysate-to-plasma ratio for creatinine; Kt/V, total weekly Kt/V urea; rGFR, residual glomerular filtration rate; Ca, serum calcium; P, serum phosphorus; iPTH, intact parathyroid hormone*.

### Associations Between Nutritional Parameters, Inflammatory Factors, and Dialysis Adequacy

The associations between nutritional and inflammatory parameters and PD adequacy are displayed in Table 2. It is noticeable that patients in the inadequacy dialysis group were characterized with higher BMIs and UAs along with lower levels of Hb, nPCR, and TC. The subgroup analysis revealed that the overweight group had a higher proportion of patients with inadequate dialysis than the other groups (*p* < 0.001). However, we found no significant differences in Alb, PA, or TRF levels between groups. As for the inflammatory factors, dialysis inadequacy patients had significantly higher hsCRP and IL-6 levels.

**Table 2:** Nutritional parameters in CAPD patients grouped by Kt/V.

**Patient characteristics**	**Total**	**Dialysis inadequacy group**	**Dialysis adequacy group**	***p*-value**
BMI (kg/m^2^)	22.32 ± 3.69	24.15 ± 3.95	21.63 ± 3.33	<0.001*
**BMI group [*****n*** **(%)]**				
<18.5	38	5 (6.8)	33 (17.1)	<0.001^#^
18.5–24.0	152	31 (42.5)	121 (62.7)	<0.001^#^
≥24.0	76	37 (50.7)	39 (20.2)	
Hb (g/dL)	103.7 ± 17.5	99.9 ± 18.4	105.0 ± 17.0	0.031*
Alb (g/dL)	37.8 ± 5.6	38.0 ± 5.3	37.7 ± 5.7	0.619
nPCR (g/kg/day)	0.93 ± 0.23	0.79 ± 0.18	0.98 ± 0.22	<0.001*
TC (mmol/L)	4.37 ± 1.11	4.17 ± 1.19	4.45 ± 1.08	0.066
PA (g/dL)	372.2 ± 87.7	377.8 ± 98.0	370.2 ± 83.7	0.532
TRF (g/L)	1.8 (1.6–2.1)	1.7 (1.6–2.0)	1.9 (1.7–2.2)	0.088
UA (μmol/L)	387.5 (345.0–438.0)	401.0 (367.0–465.7)	378.0 (338.0–429.5)	0.003*
TG (mmol/L)	1.34 (0.96–2.17)	1.37 (1.04–2.21)	1.32 (0.93–2.16)	0.609
hsCRP (mg/L)	1.9 (0.4–6.5)	3.5 (0.6–5.8)	1.7 (0.4–5.2)	0.025*
IL-6 (pg/mL)	5.2 (3.0–8.8)	6.4 (4.2–11.0)	4.8 (2.9–8.3)	0.024*

Data presented as median (first-third interquartile range) or mean ± SD or number (percentage). *Stands for p < 0.05, ^#^p < 0.00 compared to BMI ≥ 24.0 (kg/m^2^) group.

*BMI, body mass index; PA, Prealbumin; Alb, Albumin; nPCR, normalized protein catabolic rate; Hb, hemoglobin; UA, uric acid; TC, total cholesterol; TG, triglycerides; hsCRP, high-sensitivity C-reactive protein; IL-6, interleukin-6*.

### Impact of Primary Cause of ESRD on Dialysis Adequacy

Further analyses were performed to determine the impact of the patients' primary cause of ESRD on their dialysis adequacy, nutritional, and inflammatory statuses (Supplementary Table 1). DM patients featured the lowest levels of nPCR and rGFR, as well as the highest levels of BMI, hsCRP, IL-6, and leak-protein. Interestingly, the diabetic patients also presented with a significantly higher proportion of H and HA peritoneal transport status. These results illustrate that the diabetic patients in this study had more severe inflammation, worse residual renal function, and poorer nutritional conditions.

### Correlation Analyses and Risk Factors Associated With Dialysis Adequacy

In the correlation analyses, several important factors were found to be related to dialysis adequacy in CAPD patients (Table 3), such as HA or H transport status, DM, GN, rGFR, Hb, Ca, leak-protein, P, and UA. For the nutritional markers, dialysis adequacy was most closely associated with nPCR and BMI. Meanwhile, the inflammatory markers hsCRP and IL-6 were negatively correlated to dialysis adequacy. For ease of analysis and interpretation, we transformed nPCR and rGFR into binary variables. Using the ROC curve for each, the cut-off values of nPCR and rGFR that best distinguished dialysis adequacy were 0.815 and 2.43, respectively. We next applied a multivariable step forward regression analysis including all of the factors that were significant on univariate analysis to explore risk factors. The analysis revealed that gender (male), nPCR < 0.815 g/kg/day, higher weight, and rGFR < 2.43 mL/min/1.73 m^2^ were the strongest independent risk factors for dialysis inadequacy in CAPD patients (Table 4).

**Table 3:** Correlation analysis of factors associated with dialysis adequacy in CAPD patients.

**Variable**	***r***	***P-*value**
Gender (M)	−0.387	<0.001*
DM	−0.209	0.001*
GN	0.130	0.038*
HTN	−0.031	0.618
H& HA	−0.194	0.002*
nPCR (g/kg/day)	0.391	<0.001*
UA (μmol/L)	−0.183	0.003*
Leak-protein (g/day)	−0.139	0.023*
Hb (g/L)	0.127	0.039*
P (μmol/L)	−0.269	<0.001*
Ca (μmol/L)	0.176	0.004*
hsCRP (mg/L)	−0.138	0.025*
IL-6 (pg/mL)	−0.139	0.023*
rGFR (mL/min/1.73 m^2^)	0.267	<0.001*
BMI (kg/m^2^)	−0.303	<0.001*

* *Stands for p < 0.05*.

*DM, diabetes mellitus; HTN, hypertensive nephrosclerosis; GN, glomerulonephritis; HA, high average; H, high; nPCR, normalized protein catabolic rate; UA, uric acid; Hb, hemoglobin; P, serum phosphorus; Ca, serum calcium; hsCRP, high-sensitivity C-reactive protein; IL-6, interleukin-6; rGFR, residual glomerular filtration rate; BMI, body mass index*.

**Table 4:** Multivariate logistic model for dialysis adequacy in CAPD patients.

**Variable**	**OR**	**95%CI**		***p-*value**
		**Lower**	**Upper**	
Gender (male)	0.082	0.037	0.182	<0.001*
nPCR < 0.815 g/kg/day	0.227	0.103	0.500	<0.001*
rGFR < 2.43 mL/min/1.73 m^2^	0.114	0.047	0.273	<0.001*
Overweight	0.458	0.209	1.001	0.050*

* *Stands for p ≤ 0.05*.

### Effect of nPCR on Prediction of Dialysis Adequacy

**Figure 2:**
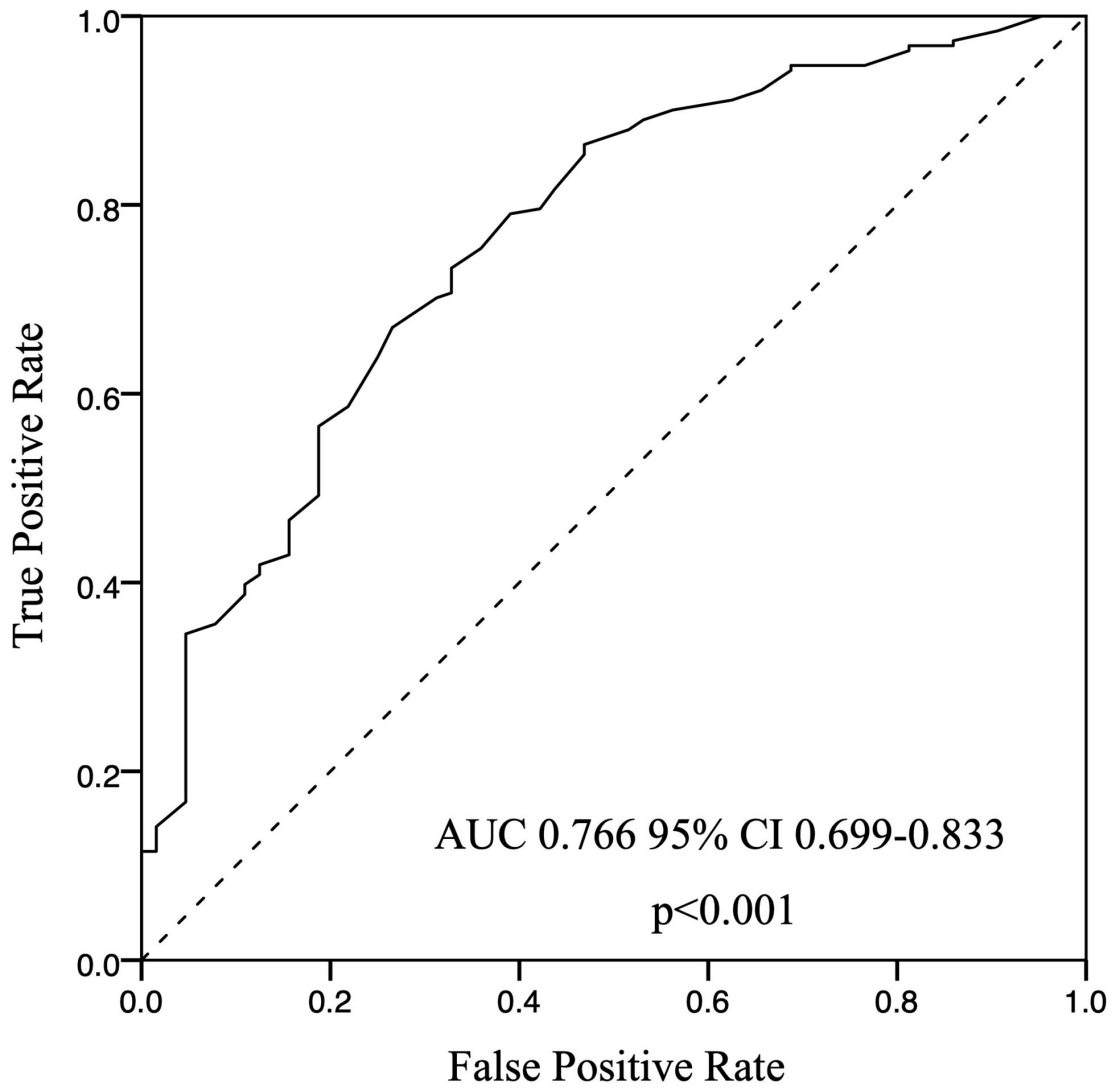
ROC curve for nPCR, the cut-off point 0.815 (g/kg/day), *p* < 0.001.

To investigate the predictive power of nPCR in evaluating dialysis adequacy (Figure 2), we determined the best cut-off for nPCR-based prediction using a ROC analysis. The ROC analysis further revealed that the nPCR cut-off of 0.815 g/kg/day, using Youden's index had a sensitivity of 79.3%, a specificity of 60.3%, and an area under the ROC curve of 0.766 (0.699–0.833) (Figure 2).

## Discussion

Dialysis adequacy is recognized as a crucial factor affecting prognosis in CAPD patients (2). The prevention of inadequate dialysis is therefore central in effectively treating such patients, yet the factors related to dialysis adequacy are ambiguous, and the relationship between nutrition, especially markers of nutrition, and dialysis adequacy remains controversial (3, 4). In light of the limited research in this area, we conducted this study and found that nPCR was not only an important marker of nutrition, but also closely related to CAPD adequacy.

In our study, dialysis adequacy was significantly correlated with nPCR. We observed a significantly higher level of nPCR in the dialysis adequacy group. Considering that nPCR and rGFR are strongly correlated with dialysis adequacy and finding the optimal cut-off value for clinical intervention, we transformed them into binary variables. Further analysis manifested that nPCR ≥ 0.815 g/kg/day was closely associated with dialysis adequacy in our study. Indeed, this is the first large CAPD study to verify the clear association between nPCR and dialysis adequacy. Moreover, our cut-off value of 0.815 is consistent with previous studies suggesting that nPCR values lower than 0.8 g/kg/day represent poor nutrition in dialysis patients (5), and that nutritional status is closely related to dialysis adequacy.

In the current literature, nPCR is considered a useful measure for assessing dietary protein intake (7), and values of nPCR < 0.8 g/kg/day are defined as a marker of poor nutrition status according to the KDOQI guidelines and other authorities (5, 8). Previous studies have placed attention on nPCR as a nutritional marker and its overall effect on morbidity and mortality, so the strong association between malnutrition and outcome in this population is well-established, while knowledge of its specific relationship with dialysis adequacy is limited (9, 11). In our study, nPCR < 0.815 g/kg/day was identified as an independent risk factor for dialysis inadequacy. There may exist a physiologic association between nutritional status and CAPD adequacy. On one hand, the intake of protein depends on the dose of dialysis, where patients who suffer from anorexia due to the influence of uremic toxicity will have reduced dietary protein and energy intake resulting from the inadequate dialysis and thus become predisposed to malnutrition (12). On the other hand, there is also a strong association between malnutrition and inflammation in dialysis patients. Malnutrition could thus aggravate dialysis inadequacy by stimulating persistent inflammation (13).

In the present study, we confirmed the general correlation between nutritional markers and dialysis adequacy, and found a definite correlation with nPCR alone. No significant differences were seen in traditional nutritional markers such as Alb, PA, TRF, or TG values. Further analysis revealed that only nPCR was an independent risk factor for dialysis inadequacy in CAPD patients, rather than Alb and PA. In fact, the relationship between albumin and dialysis adequacy remains controversial, in part due to the fact that it is both a nutritional and a biochemical marker. It is therefore difficult to use albumin as an isolated nutritional marker given its direct relationship with inflammation. Another important factor to be considered is hypoalbuminemia variability, which can be influenced by the hydration status of the patient, malnutrition, synthesis reduction, and organic stress, among other factors (6, 7, 14, 15). In consideration of the fact that poor dietary intake is most often the critical etiology for malnutrition, and one advantage of nPCR is its accuracy in monitoring protein intake, we speculate that nPCR is more accurate and sensitive than traditional nutrition markers for the prediction of dialysis adequacy in CAPD patients.

Although we found that inflammatory factors were associated with dialysis inadequacy in univariate analyses, we found no significant associations in multivariate analyses, in accordance with previous findings (16). We speculate that such findings might be explained by the fact that inflammation status is closely related to the levels of nPCR, resulting in the appearance of an association with dialysis adequacy. Further studies are needed. Of note, our other findings such as male gender and higher weight as risk factors for CAPD adequacy, are consistent with earlier studies (17). The mechanisms underlying our findings may be related to the fact that Kt/V is strongly influenced by body surface area, fat mass, muscle mass, and composition (BMI, weight, and height). Indeed, these indices and body composition in general are different in males and females. Males also tend to produce more metabolites due to their larger body index, higher basal metabolism, and larger dietary intake, which combined with limited peritoneal clearance and residual renal function; these metabolites are not effectively cleared as they are in females, resulting in inadequate dialysis (17–19). However, the most recent ISPD guidelines set the target Kt/V level in dialysis patients as constant regardless of the patient's gender (8). Owing to the gender difference observed in our study and others, it is thought provoking to consider whether gender should be taken into account when setting the target Kt/V level in future guidelines. In addition, we found that decreased rGFR was also a predictor of inadequate dialysis, in accordance with the CANUSA study (20) and other previous investigations (21). To our knowledge, rGFR represents residual renal function (RRF). Therefore, decreased rGFR indicates the compromised clearance of metabolic products as well as volume load (12, 22), which could aggravate the risk of dialysis inadequacy.

Interestingly, it is worth considering that the primary cause of ESRD was also related to dialysis adequacy. In our study, diabetic patients had a significantly higher proportion of inadequate dialysis. DM is already a well-known risk factor for protein-energy wasting and plays a significant role in the production of inflammatory cytokines, leading to peritoneal membrane sclerosis and vasculopathy as well as higher protein loss from the peritoneum (23). In addition, it has been reported that patients on CAPD with higher peritoneal transport rates do not attain appropriate ultrafiltration and display greater peritoneal protein loss (24). Taken together, we speculate that increased systemic inflammatory reactivity, poorer nutrition status, and H and HA peritoneal membrane permeability in DM patients drive the inadequate dialysis status in these patients.

A cross-sectional study like the current one can be helpful in assessing the implications and usefulness of different indices. Nonetheless, given that this was a single center study with a limited number of patients, our findings should be interpreted within the context of these limitations. Since we only enrolled CAPD patients in China, our results may not necessarily extend to other patients. In addition, due to the nature of its cross-sectional design, the study lacked long-term morbidity, technical failure, and mortality data. The relationship between nPCR and long-term prognosis is therefore unclear. A well-designed prospective study is needed to provide further verification and understanding of the current findings.

## Conclusion

CAPD patients with dialysis inadequacy also suffer from a more severe inflammatory status and poorer nutritional status. The current study indicates that gender (male), nPCR < 0.815 g/kg/day, higher weight, and rGFR < 2.43 mL/min/1.73 m^2^ are risk factors for inadequate dialysis. Among the nutritional markers, nPCR may be a superior nutritional marker to indicate dialysis adequacy.

## Data Availability Statement

The raw data supporting the conclusions of this article will be made available by the authors, without undue reservation.

## Ethics Statement

The study was approved by the Medical Ethical Committee of West China Medical School, Sichuan University. The patients/participants provided their written informed consent to participate in this study. Written informed consent was obtained from the individual(s) for the publication of any potentially identifiable images or data included in this article.

## Author Contributions

AQ, YT, and WQ: conception, design, and manuscript writing. AQ, XY, HZ, and XL: administrative support. AQ, YT, HZ, XL, and WQ: collection and assembly of data. AQ, XY, HZ, XL, YT, and WQ: data analysis and interpretation. AQ, YT, and WQ: final approval of manuscript. All authors contributed to the article and approved the submitted version.

## Conflict of Interest

The authors declare that the research was conducted in the absence of any commercial or financial relationships that could be construed as a potential conflict of interest. The reviewer ZH declared a shared affiliation, though no other collaboration with the authors to the handling Editor.
