# Supplementary material for: Normalized Protein Catabolic Rate Is a Superior Nutritional Marker Associated With Dialysis Adequacy in Continuous Ambulatory Peritoneal Dialysis Patients
Source: Front Med (Lausanne). 2021 Jan 12;7:603725. doi: 10.3389/fmed.2020.603725 (PMC7835658; doi:10.3389/fmed.2020.603725)
Supplement: Supplementary file 1 [file Table_1.docx]

**Supplementary Table 1:** Comparison of primary cause of ESRD in CAPD patients

| patient characteristics | GN | DM | HTN |
| --- | --- | --- | --- |
| Patients (n) | 144 | 35 | 53 |
| Peritoneal transport type  H&HA [n (%)]  L&LA [n (%)]  Age (years) | 103 (71.5) *  41 (28.5) *  45.8±12.6* | 33 (94.3)  2 (5.7)  59.8±10.1 | 177 (76.3) *  55 (23.7) *  53.7±12.3*^#^ |
| nPCR(g/kg/day) | 0.95±0.23* | 0.85±0.21 | 0.86±0.20*^#^ |
| Kt/V | 2.1±0.5* | 1.7±0.4 | 1.9±0.6* |
| Alb(g/L） | 37.5±6.2 | 37.0±4.2 | 37.8±3.9 |
| PA(mg/L） | 367.7±89.0 | 337.3±86.3 | 365.9±76.1 |
| Hb (g/L) | 102.9±17.2 | 104.2±12.6 | 104.2±17.4 |
| BMI (kg/m^2^) | 21.3±3.3* | 24.7±3.8 | 23.9±3.7^#^ |
| IL-6 (pg/mL) | 4.32(2.78-7.85) * | 10.2(7.2-17.6) | 5.84(3.89-9.91) * |
| TRF(g/L) | 1.83(1.59-2.14) | 1.83(1.65-2.12) | 1.87(1.69-2.13) |
| hsCRP (g/L) | 1.36(0.34-5.19) * | 6.97(1.96-14.60) | 3.85(0.81-9.65) |
| leak-protein (g/day) | 5.28 (4.20-7.02) * | 7.72 (5.35-7.30) | 5.86 (4.20-7.37) * |
| rGFR (mL/min/1.73m^2^) | 1.14(0.00-3.55) | 0.39(0-1.67) | 1.68(0.25-4.37) * |

**Note:** Data presented as median (first-third interquartile range) or mean ±SD or number (percentage) **p* < 0.05 compared to DM group; ^#^*p* < 0.05 compared to GN group.

**Abbreviations:** L, low; LA, low average; HA, high average; H, high; DM, diabetes mellitus; HTN, hypertensive nephrosclerosis; GN, glomerulonephritis; PA, Prealbumin; Alb, Albumin; TRF, transferrin; Hb, hemoglobin; BMI, body mass index; IL-6, interleukin-6; hsCRP, high-sensitivity C-reactive protein; nPCR, normalized protein catabolic rate; rGFR, residual glomerular filtration rate
